# A physiological role of cyclic electron transport around photosystem I in sustaining photosynthesis under fluctuating light in rice

**DOI:** 10.1038/srep20147

**Published:** 2016-02-02

**Authors:** Wataru Yamori, Amane Makino, Toshiharu Shikanai

**Affiliations:** 1 Center for Environment, Health and Field Sciences, Chiba University, 6-2-1 Kashiwa-no-ha, Kashiwa, Chiba 277-0882, Japan; 2 Department of Applied Plant Science, Graduate School of Agricultural Science, Tohoku University, 1-1 Tsutsumidori-Amamiyamachi, Aoba-ku, Sendai 981-8555, Japan; 3 Department of Botany, Graduate School of Science, Kyoto University, Sakyo-ku, Kyoto 606-8502, Japan; 4 PRESTO, Japan Science and Technology Agency (JST), 4-1-8 Honcho, Kawaguchi, Saitama 332-0012, Japan; 5 CREST, JST, 4-1-8 Honcho, Kawaguchi, Saitama 332-0012, Japan

## Abstract

Plants experience a highly variable light environment over the course of the day. To reveal the molecular mechanisms of their photosynthetic response to fluctuating light, we examined the role of two cyclic electron flows around photosystem I (CEF-PSI)—one depending on PROTON GRADIENT REGULATION 5 (PGR5) and one on NADH dehydrogenase-like complex (NDH)—in photosynthetic regulation under fluctuating light in rice (*Oryza sativa* L.). The impairment of PGR5-dependent CEF-PSI suppressed the photosynthetic response immediately after sudden irradiation, whereas the impairment of NDH-dependent CEF-PSI did not. However, the impairment of either PGR5-dependent or NDH-dependent CEF-PSl reduced the photosynthetic rate under fluctuating light, leading to photoinhibition at PSI and consequently a reduction in plant biomass. The results highlight that (1) PGR5-dependent CEF-PSI is a key regulator of rapid photosynthetic responses to high light intensity under fluctuating light conditions after constant high light; and (2) both PGR5-dependent and NDH-dependent CEF-PSI have physiological roles in sustaining photosynthesis and plant growth in rice under repeated light fluctuations. The highly responsive regulatory system managed by CEF-PSI appears able to optimize photosynthesis and plant growth under naturally fluctuating light conditions.

Plants experience a highly variable light environment over the course of the day on timescales of seconds, minutes, or hours owing to changes in leaf angle, cloud cover, and canopy cover^1^. When light intensity exceeds the ability of leaves to use the light in photosynthesis, the excess light energy can lead to the formation of reactive oxygen species (ROS), and eventually to photoinhibition. Thus, plants need a highly responsive regulatory system to keep photosynthetic light reactions in balance with the needs and restrictions of the downstream metabolism. Efficient utilization of light energy through an optimized photosynthetic response under fluctuating light conditions is of ecological and agronomic interest.

Photosynthesis starts with the absorption of light by the light-harvesting systems, which drive photosynthetic electron transport through the thylakoid membranes of the chloroplasts^2^. Electrons derived from the splitting of water in photosystem II (PSII) ultimately reduce NADP^+^ to NADPH via photosystem I (PSI). This linear electron transport passes through the cytochrome (Cyt) *b*_*6*_*/f* complex, generating a proton gradient across the thylakoid membrane (ΔpH). Together with the protons deposited in the thylakoid lumen by the water-splitting complex associated with PSII, the protons translocated at the Cyt *b*_*6*_*/f* complex into the lumen enable ATP production by chloroplastic ATP synthase. NADPH and ATP generated by light reactions are then utilized in the Calvin–Benson cycle and other assimilatory reactions. The cyclic electron flow around PSI (CEF-PSI), which also passes through the Cyt *b*_*6*_*/f* complex, can also generate a ΔpH across the thylakoid membrane, and also produces ATP but without any accumulation of NADPH in chloroplasts^2^.

CEF-PSI consists of two partly redundant pathways: the main pathway depends on the PGR5 (PROTON GRADIENT REGULATION 5) and PGRL1 (PGR5-LIKE PHOTOSYNTHETIC PHENOTYPE 1) proteins^3^,^4^, whereas the minor pathway is mediated by the chloroplast NADH dehydrogenase-like (NDH) complex^5^,^6^. Most likely, CEF-PSI involving PGR5 and PGRL1 proteins mainly mediates electron transport from ferredoxin to plastoquinone, contributes to ΔpH formation in photosynthesis, and contributes to ATP supply for CO_2_ fixation^2^,^4^. The Arabidopsis *pgr5* mutant grows similarly to the wild-type (WT) plants under constant low-light conditions, but is sensitive to constant high-light conditions, especially under low CO_2_ concentration^7^. Recent studies showed that fluctuating light induced photodamage in PSI and severely retarded the growth in *pgr5* plants^8^,^9^,^10^. These results indicate that PGR5 is essential for survival under field conditions in Arabidopsis. In contrast, *PGR5*-knockdown (KD) rice lines showed a mild decline in CO_2_ assimilation and biomass production, although levels of ΔpH formation were decreased and the redox state of P700 were severely reduced^11^. Although the molecular function of PGR5 is likely conserved among plants, its physiological contribution to the regulation of photosynthesis might differ between Arabidopsis and rice.

On the other hand, chloroplast NDH forms a supercomplex with PSI and recycles electrons from ferredoxin to plastoquinone and subsequently to PSI^2^. NDH-defective tobacco and Arabidopsis mutants did not show any decrease in photosynthesis or growth under moderate growth conditions^5^,^12^. In addition, NDH-knockout Arabidopsis mutants did not show any growth suppression or reduction of photosynthetic performance even under fluctuating light, leading to the conclusion that NDH dependent CEF-PSI does not contribute to the response to fluctuating light^9^,^10^. However, NDH-deficient mutants showed mild sensitivity to various stresses, including strong light^13^, high temperature^14^, and low temperature^15^. These suggest that chloroplast NDH could act to alleviate oxidative stress in chloroplasts under excessive light^2^. However, the mutant phenotypes tested are mild, and the mechanism by which chloroplast NDH might alleviate oxidative stresses is unclear because of the low rate of electron transport monitored *in vivo* and in isolated thylakoids^16^,^17^. The clear phenotype of NDH-deficient mutants is observed only when the PGR5-PGRL1 protein-dependent pathway is also impaired in double mutants^12^, indicating that chloroplast NDH may act as a safety valve when the stroma is highly reduced. However, the physiological function of NDH-dependent CEF around PSI remains to be clarified.

Since abiotic stresses limit crop productivity, understanding the physiological processes that underlie stress injury and the mechanisms of plant tolerance to the stresses is of immense importance to agriculture. The photosynthetic characteristics brought about by acclimation or adaptation to growth light levels have been studied intensively. Many plants acclimate to their growth light environment and alter their biochemical composition and morphology of leaves and whole plants^18^,^19^,^20^. Dynamic fluctuation of light intensity has recently become a subject of experimental research. However, the molecular mechanisms of photosynthetic responses to fluctuating light remain to be clarified. Here, we examined the physiological role of PGR5-dependent and NDH-dependent CEF-PSI in photosynthetic regulation and plant growth under fluctuating light in rice (*Oryza sativa* L.), a high-light-adaptive major crop. We measured gas exchange, chlorophyll fluorescence, and P700^+^ reduction rate under fluctuating light. The results clearly show that both PGR5-defective and NDH-defective mutants suffered under fluctuating light, with PSI as the primary target of photodamage, and had reduced plant growth. Therefore, not only PGR5-dependent but also NDH-dependent cyclic electron transport is essential for photoprotection of PSI under fluctuating light in rice.

## Results

### Photosynthetic components in PGR5-defective and NDH-defective mutants

When plants were grown at a constant light intensity of 500 μmol photons m^–2^ s^–2^ with a 14-h photoperiod, contents of leaf nitrogen, rubisco, and chlorophyll per unit leaf area and the chlorophyll-*a/b* ratio were similar among WT plants, *PGR5* KD plants (defective in PGR5-dependent CEF-PSI pathway), and *crr6* mutant plants (defective in NDH-dependent CEF-PSI pathway) (Table 1). A previous study confirmed that levels of PsaA (PSI reaction center), PsbA (PSII reaction center), and PsbS (pH sensor for qE induction) were unaffected in the *PGR5* KD lines in rice^11^. We therefore consider that alterations in photosynthetic properties as a result of light treatments would primarily be the result of the stress due to defects in PGR5 and CRR6, rather than general growth defects.

### Role of PGR5- and NDH-dependent cyclic electron transport in photosynthetic response under fluctuating light after constant high light

**Figure 1:**
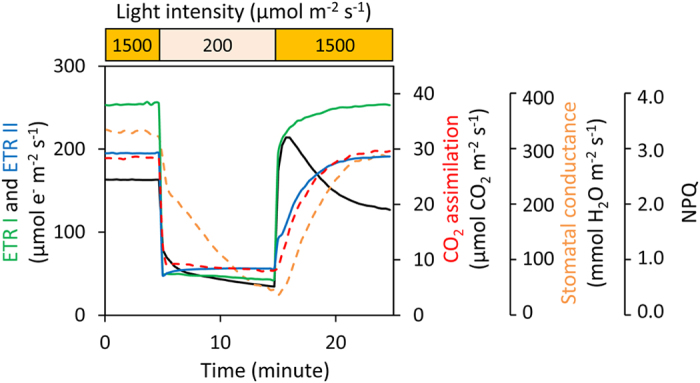
Responses of photosynthetic parameters after changes in light intensity in rice WT plants (*Oryza sativa* cv. Nipponbare). We simultaneously measured the electron transport rate around photosystem I (ETR I) and around photosystem II (ETR II), CO_2_ assimilation rate at a CO_2_ concentration of 400 μmol mol^–1^, stomatal conductance, and non-photochemical quenching (NPQ). Leaves were first allowed to reach a steady rate of photosynthesis at 1500 μmol photons m^–2^ s^–1^ for 30 min. The photosynthetic parameters were recorded for 5 min, then the light intensity was reduced to 200 μmol photons m^–2^ s^–1^ for 10 min, and then returned to 1500 μmol photons m^–2^ s^–1^ for 10 min. Values are means, *n* = 5 or 6.

We simultaneously measured gas exchange, chlorophyll fluorescence, and P700 redox state in WT plants under the initial cycle of fluctuating light after constant high light (Fig. 1). The electron transport rate through PSI (ETR I) and through PSII (ETR II) were estimated from Y(I) and Y(II), respectively, on the assumption that there are no changes in the accumulating ratio of PSI to PSII or their antenna sizes (see, Materials & Methods). High light after 10 min at low light increased ETR I and transiently induced non-photochemical quenching (NPQ), probably via the activation of CEF-PSI. Subsequently, ETR II was increased as NPQ relaxed. Then the CO_2_ assimilation rate increased with stomatal conductance.

**Figure 2:**
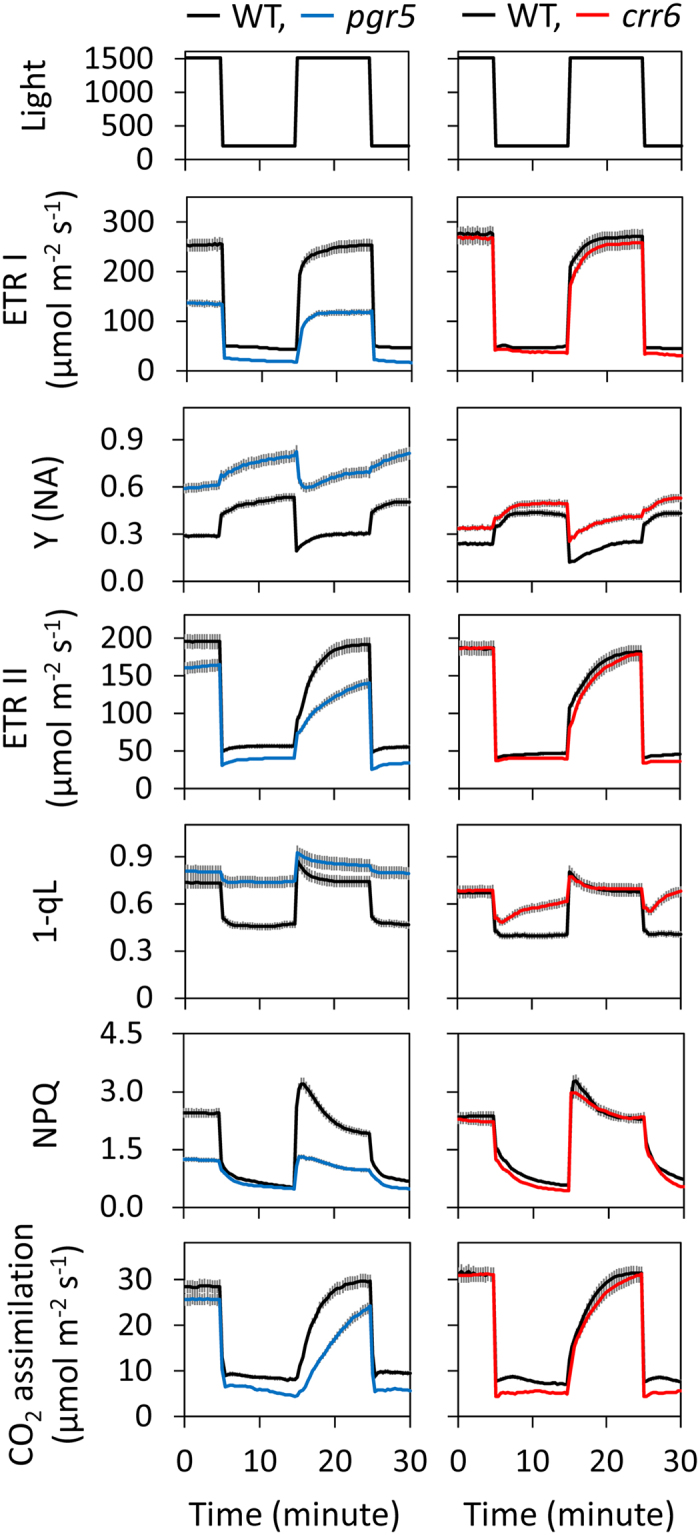
Time course of photosynthetic responses after changes in light intensity in *PGR5*-knockdown plants, *crr6* mutant plants, and WT plants. The same fluctuating light regime as in Fig. 1 was used. We simultaneously measured the electron transport rate around PSI (ETR I) and around PSII (ETR II), CO_2_ assimilation rate at a CO_2_ concentration of 400 μmol mol^–1^, the fraction of PSI reaction centers that are closed owing to acceptor side limitation (Y(NA)), the redox state of the plastoquinone pool (1-qL), and non-photochemical quenching (NPQ). The graphs compare *PGR5* KD plants with their WT (*Oryza sativa* cv. Nipponbare), and *crr6* mutant plants with their WT (*Oryza sativa* cv. Hitomebore). Values are means ± SE, *n* = 5 or 6.

Next, we analyzed the contribution of the two CEF-PSI pathways to photosynthetic response under the first two cycles of fluctuating light after constant high light in *PGR5* KD plants and *crr6* mutant plants. During the high-light phase, the responses of ETR I, ETR II, and CO_2_ assimilation rate at a CO_2_ concentration of 400 μmol mol^–1^ were virtually identical between the *crr6* mutants and the WT, whereas the fraction of PSI reaction centers that are closed due to acceptor side limitation, Y(NA), was significantly higher in the *crr6* mutants (Fig. 2, Supplemental Fig. 1). On the other hand, during the low-light phase, the *crr6* mutants had a lower NPQ and increased reduction (redox state) of the plastoquinone pool (high 1-qL) compared to the WT plants. ETR I, ETR II, and CO_2_ assimilation rate responded to an increase in light intensity much more slowly in the *PGR5* KD plants than in the WT plants. The *PGR5* KD plants could not build up NPQ during the high-light phase, and showed higher Y(NA) and 1-qL during both high-light and low-light phases. These results indicate that PGR5-dependent CEF-PSI is a key regulator of the rapid photosynthetic response to high light intensity under fluctuating light after constant high light, but the contribution of NDH-dependent CEF-PSI is small.

### Role of PGR5- and NDH-dependent cyclic electron transport in photosynthesis under repeated light fluctuations

**Figure 3:**
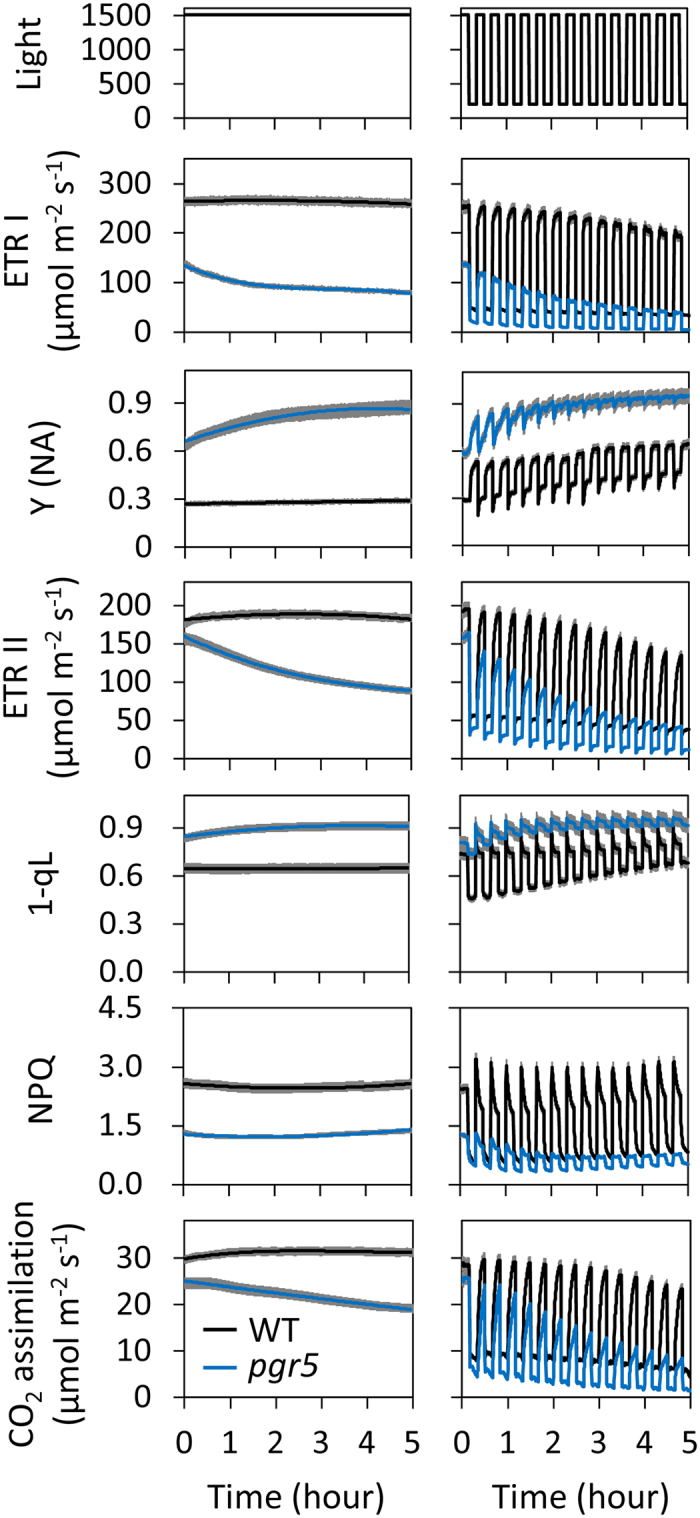
Responses of photosynthetic parameters to either constant high-intensity or fluctuating light in *PGR5*-knockdown and WT (‘Nipponbare’) plants. Photosynthetic parameters were monitored at a CO_2_ concentration of 400 μmol mol^–1^ under either constant high-intensity light (1500 μmol photons m^–2^ s^–1^) or fluctuating light (200 μmol m^–2^ s^–1^ for 10 min and 1500 μmol m^–2^ s^–1^ for 10 min) for 5 h. Abbreviations are the same as in Fig. 2. The responses of the quantum yield of PSI (Y(I)) and PSII (Y(II)) to fluctuating light were presented in Supplemental Fig. 3. Values are means ± SE, *n* = 5 or 6.

**Figure 4:**
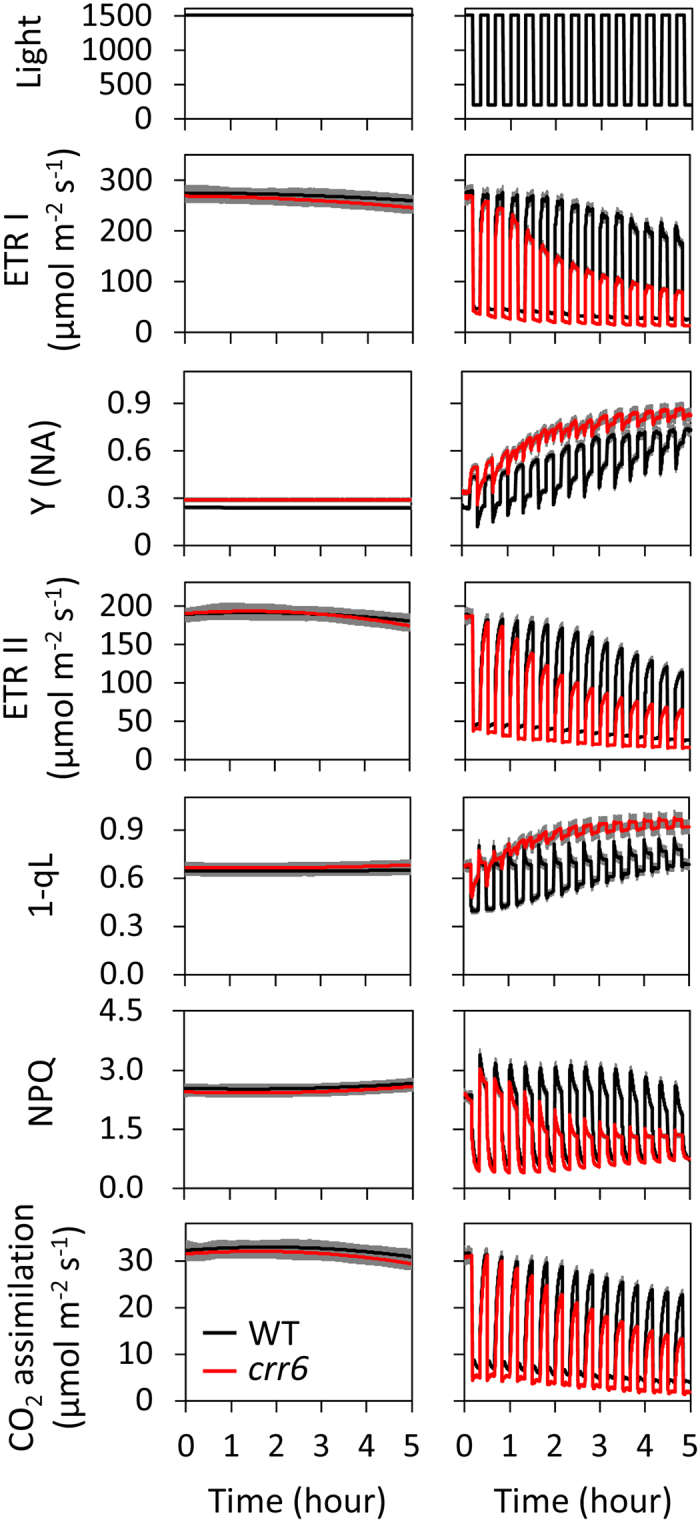
Responses of photosynthetic parameters to either constant high-intensity or fluctuating light in *crr6* mutant and WT (‘Hitomebore’) plants. Photosynthetic parameters were monitored at a CO_2_ concentration of 400 μmol mol^–1^ under either constant high-intensity light (1500 μmol photons m^–2^ s^–1^) or fluctuating light (200 μmol m^–2^ s^–1^ for 10 min and 1500 μmol m^–2^ s^–1^ for 10 min) for 5 h. Abbreviations are the same as in Fig. 2. The responses of the quantum yield of PSI (Y(I)) and PSII (Y(II)) to fluctuating light were presented in Supplemental Fig. 4. Values are means ± SE, *n* = 5 or 6.

In WT plants, under constant high light, ETR I, ETR II, and CO_2_ assimilation rate were constant over 5 h (Figs 3 and 4). On the other hand, under fluctuating light, ETR I, ETR II, and CO_2_ assimilation rate during both low-light and high-light phases gradually decreased over 5 h. This decrease in both cultivars (‘Hitomebore’ and ‘Nipponbare’) indicates that the light fluctuation is stressful even for WT plants (Supplemental Fig. 2). The saturating pulses applied every 20 s during the measurements may have enhanced the sensitivity to high light to some extent.

Next, we evaluated the impact of the defects in PGR5- and NDH-dependent CEF-PSI on whole-chain electron transport under fluctuating light. Under constant high light, the *PGR5* KD caused a large reduction in ETR I but a smaller reduction in ETR II, and concomitant increases in 1-qL and Y(NA), and a reduction in NPQ (Fig. 3). Consequently, CO_2_ assimilation rate was decreased to the same extent as ETR II in the *PGR5* KD plants. Under constant light, ETR I, ETR II, and CO_2_ assimilation rate gradually decreased in the *PGR5* KD plants over 5 h. Under fluctuating light, they decreased in a stepwise manner at every transition to low light, a pattern not seen in the WT plants (Fig. 3). The redox state of the plastoquinone pool (1-qL) of the *PGR5* KD plants was increased, whereas NPQ was suppressed, during both low-light and high-light phases. The quantum yield of PSI (Y(I)) and PSII (Y(II)) showed a similar trend with ETR I and ETR II, respectively (Supplemental Fig. 3). The results indicate that during the high-light phases, the photosynthetic electron transport system accumulates reducing power that cannot be dissipated during the subsequent low-light phases, thus progressively decreasing the PSII yield and increasing the redox state of the plastoquinone pool in *PGR5* KD plants.

Under constant high light in the *crr6* mutants, ETR I, ETR II, CO_2_ assimilation rate, and the other photosynthetic parameters (1-qL, NPQ, Y(NA)) were constant over 5 h, and were virtually identical to those in the WT plants (Fig. 4). Under fluctuating light, ETR I, ETR II, and CO_2_ assimilation rate decreased in a stepwise manner at every transition to low light in the *crr6* mutant (Fig. 4), as in the *PGR5* KD plants (Fig. 3). In contrast, in the *crr6* mutants, 1-qL and Y(NA) increased in a stepwise manner at every transition to low light and were higher than in WT plants. As a result, photosynthetic capacity (i.e., ETR I, ETR II, CO_2_ assimilation rate) progressively decreased as the redox state of the plastoquinone pool increased, because the photosynthetic electron transport system of the *crr6* plants accumulated reducing power that could not be dissipated before the subsequent high-light phases. This was supported by the data that Y(I) and Y(II) also showed a similar trend with ETR I and ETR II, respectively (Supplemental Fig. 4).

Stomatal conductance under fluctuating light was not significantly affected in either mutant line (Supplemental Fig. 5). These results suggest that the reduction in CO_2_ assimilation rate under the light fluctuations was due to the reduction in photosynthetic capacity due to the lack of PGR5- and NDH-dependent CEF-PSI.

### Role of PGR5- and NDH-dependent cyclic electron transport in the alleviation of photoinhibition under fluctuating light conditions

**Figure 5:**
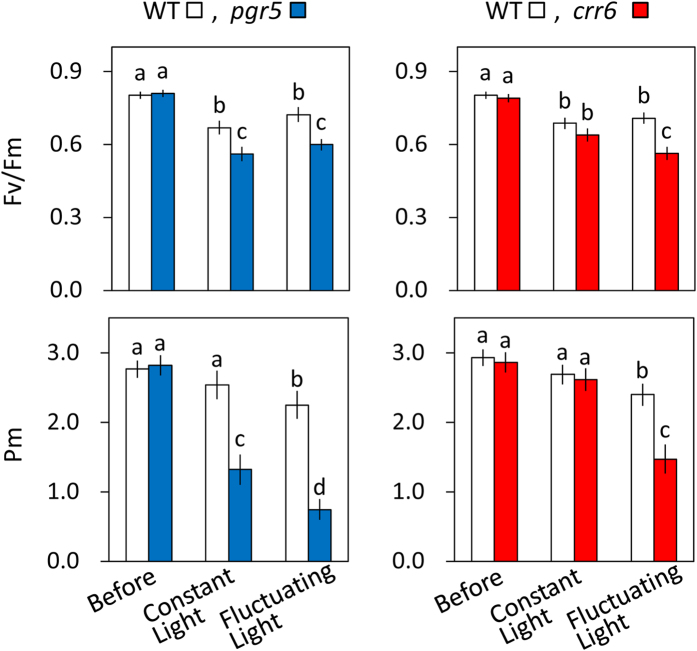
Effect of *PGR5* knockdown or *crr6* defect on alleviation of photoinhibition. The maximum level of the P700 signal of PSI (*P*_m_, full oxidation of P700) and the maximum quantum yield of PSII (*F*_v_/*F*_m_) were measured before and after treatment with constant high-intensity or fluctuating light for 5 h. Values were subsequently measured after dark incubation for 30 min. Light treatments were the same as in Figs 3 and 4. Values are means ± SE, *n* = 5 or 6. Significant differences among *PGR5 KD* plants and the WT plants are examined by Tukey–Kramer multiple comparison test (*P* < 0.05).

We analyzed the effects of PGR5- and NDH-dependent CEF-PSI on photoinhibition. The maximum level of the P700 signal (*P*_m_, full oxidation of P700) in the dark and the maximum quantum yield of PSII (*F*_v_/*F*_m_) were measured before and after treatment with constant high light (1500 μmol photons m^–2^ s^–1^) or fluctuating light (1500 μmol m^–2^ s^–1^ for 10 min and 200 μmol m^–2^ s^–1^ for 10 min) for 5 h. Before treatment, *P*_m_ and *F*_v_/*F*_m_ were similar among *PGR5* KD, *crr6* mutant, and WT plants. In the WT plants, constant high light significantly reduced only *F*_v_/*F*_m_, whereas fluctuating light reduced *F*_v_/*F*_m_ and *P*_m_, indicating that PSII is susceptible to constant high light, whereas PSI is more susceptible to fluctuating light than to constant high light (Fig. 5). In the *PGR5* KD plants, constant high light decreased *F*_v_/*F*_m_ by 16% and *P*_m_ by 48%. Fluctuating light decreased *F*_v_/*F*_m_ by 17% and *P*_m_ by 67%. In the *crr6* mutant plants, constant high light decreased *P*_m_ and *F*_v_/*F*_m_ only slightly, indicating that chloroplast NDH does not function in the tolerance to constant high light. Fluctuating light decreased *F*_v_/*F*_m_ by 21% and *P*_m_ by 39%. Like PGR5-dependent CEF-PSI, NDH-dependent CEF-PSI is required for alleviating photodamage of both photosystems by fluctuating light.

### Role of PGR5- and NDH-dependent cyclic electron transport in plant growth under fluctuating light

**Figure 6:**
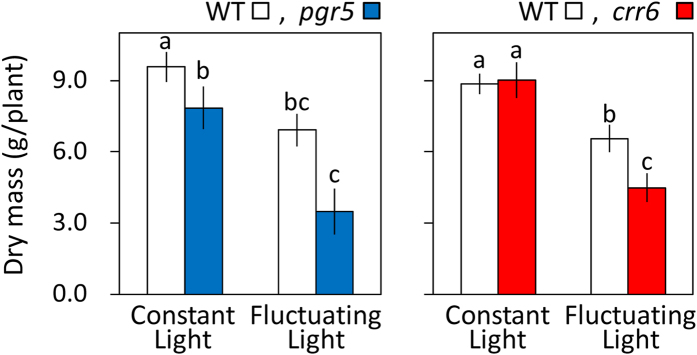
Effect of *PGR5* knockdown or *crr6* defect on plant biomass production. Plants were first grown under a constant PPFD of 500 μmol m^–2^ s^–1^ for 30 days after germination. They were then grown under either constant high-intensity light (800 μmol photons m^–2^ s^–1^) or fluctuating light (150 μmol m^–2^ s^–1^ for 10 min and 800 μmol m^–2^ s^–1^ for 10 min) for ≥50 days. The graphs compare *PGR5*-knockdown plants with their WT (‘Nipponbare’), and *crr6* mutant plants with their WT (‘Hitomebore’). Values are means ± SE, *n* = 5 or 6. Significant differences among *PGR5* KD plants, *crr6* mutant and the WT plants are examined by Tukey–Kramer multiple comparison test (*P* < 0.05).

We examined the effects of defects in PGR5- and NDH-dependent CEF-PSI on plant growth under fluctuating light. Constant light slightly suppressed growth of the *PGR5* KD plants, but fluctuating light greatly suppressed it (Fig. 6). Constant light produced similar growth in *crr6* mutant and WT plants, but fluctuating light reduced the final dry weight of *crr6* mutant plants more than that of WT plants (Fig. 6).

## Discussion

**Figure 7:**
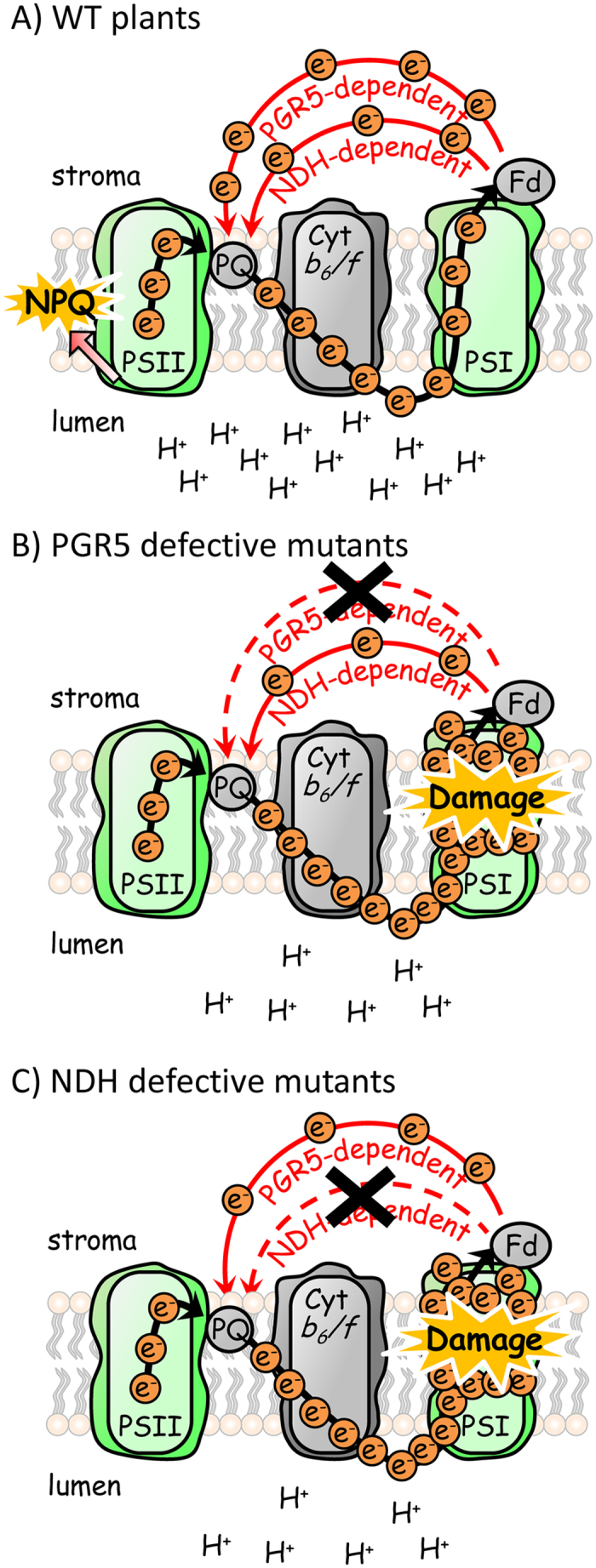
Role of PGR5-dependent and NDH-dependent CEF-PSI in photoprotection of PSI from photodamage under fluctuating light. (A) In WT plants, under fluctuating light, the electron transport system is over-reduced, and NPQ is developed via the activation of CEF-PSI, resulting in thermal dissipation of excess light energy. (B and C) In contrast, in both *PGR5*-defective and NDH-defective mutants, the electron transport system accumulates excess reducing power because it cannot dissipate heat. The cumulative strong reduction of the entire electron transport system under fluctuating light for a couple of hours would cause a strong reducing burst at the acceptor side of PSI, leading to photodamage at PSI. Thus, both PGR5-dependent and NDH-dependent CEF-PSI are essential for photoprotection of PSI under fluctuating light. Abbreviations: PSI/II, photosystem I/II; PQ, plastoquinone; Cyt *b*_*6*_*/f*, cytochrome *b*_*6*_*/f* complex; Fd, ferredoxin.

Genetic analyses using *Arabidopsis thaliana* revealed that CEF-PSI consists of two redundant pathways^2^: the main pathway, which depends on PGR5-PGRL1 proteins, and the minor pathway, which is mediated by the chloroplast NDH complex. Mutant phenotypes for CEF-PSI have been characterized only in Arabidopsis. However, the physiological role of CEF-PSI could depend on species, since evolution would have adapted the photosynthetic system to different light environments. Here, we examined the physiological consequences of defects in both PGR5 and NDH under fluctuating light in rice. The results clearly show that (1) PGR5-dependent CEF-PSI is a key regulator of a rapid photosynthetic response to high light under fluctuating light conditions, and (2) although the contribution of NDH-dependent CEF-PSI is small, both PGR5-defective and NDH-defective mutants suffer from fluctuating light, with PSI as the primary target of photodamage, and have reduced growth. Therefore, we conclude that both PGR5-dependent and NDH-dependent CEF-PSI have physiological roles in sustaining photosynthesis and growth of rice under fluctuating light (Fig. 7).

Photosynthetic induction occurring upon a sudden increase in light intensity after a prolonged period of low light or darkness has two main components based on the time scale of the processes^1^. The fast induction component is due mostly to the rapid light activation of RuBP regeneration, which is affected by photosynthetic electron transport. The second slower component is due to the light activation required for the primary carboxylation enzyme, Rubisco, combined with an increase in stomatal conductance. The mechanism of photosynthetic induction may depend on the period of low light or darkness before a sudden increase in light intensity. Our results clearly show that a sudden increase in light intensity during the fluctuating light conditions promoted ETR I and induced the rapid formation of ΔpH (i.e., NPQ) across the thylakoid membranes within 1 min (Fig. 1). Subsequently, ETR II was enhanced, with increases in CO_2_ assimilation rate and stomatal conductance. These processes need 5–10 min to be activated after illumination. Thus, it is obvious that the increase in ETR I during the first cycle of induction was faster than that of ETR II and CO_2_ assimilation rate (Fig. 1). Most likely, CEF-PSI functions immediately after a sudden increase in light intensity. In this period of photosynthetic induction, the build-up of ΔpH and the formation of NPQ would be essential not only for preventing photoinhibition, but also for activating Calvin cycle enzymes, including Rubisco activase^21^, because some enzymes of the Calvin cycle (i.e., fructose-1,6-bisphosphate phosphatase, sedoheptulose-1,7-bisphosphate phosphatase, ribulose-5-phosphate kinase, NADP-glyceraldehyde-3-phosphate dehydrogenase and Rubisco activase) are redox-regulated by thioredoxins using reduced ferredoxin produced by the electron transport reactions^22^.

Our results show that the *crr6* mutation did not affect the response of ETR I, ETR II, or CO_2_ assimilation rate during the high-light phase under fluctuating light, leading to the conclusion that NDH-dependent CEF-PSI does not affect the photosynthetic response to an increase in light intensity under fluctuating light (Fig. 2). On the other hand, the *PGR5* KD plants showed a much slower response of ETR II and CO_2_ assimilation rate following an increase in light intensity, since it caused a lower ETR I rate, and thus NPQ was suppressed immediately after irradiation. Analysis of the direct impact of NPQ on photosynthetic CO_2_ assimilation during the induction phase in transgenic rice with altered levels of PsbS showed that the accumulation of PsbS and the resulting NPQ exerts control over photosynthesis under fluctuating light^23^. Thus, we conclude that PGR5-dependent CEF-PSI and the resulting lower NPQ control the photosynthetic response during the high-light phase under fluctuating light (Fig. 2). Alternative pathways, including the water–water cycle and CEF-PSI, may kick-start photosynthesis immediately after sudden light irradiation^24^. We propose that the PGR5-dependent CEF-PSI functions mainly to start photosynthesis during the high-light phase under fluctuating light via the rapid build-up of ΔpH across the thylakoid membranes and formation of NPQ.

In WT plants, during the high-light phase of fluctuating light (on the order of minutes), the electron transport system is over-reduced (high 1-qL) and NPQ is developed (Fig. 2), resulting in the thermal dissipation of excess light energy. On the other hand, during the low-light phase, the electron transport system is oxidized (low 1-qL) and NPQ is relaxed within minutes to allow the maximum photosynthetic electron transport during subsequent high light (Fig. 2). Nevertheless, ETR I, ETR II, and CO_2_ assimilation rate showed gradual decreases over 5 h in WT plants after fluctuating light treatment (Figs 3 and 4). These plants also showed slight photoinhibition of PSI under the fluctuating light treatment (Fig. 5). Thus, PSI photoinhibition under fluctuating light could occur even in WT plants.

In contrast, the *PGR5* KD plants are deficient in the development of ΔpH across the thylakoid membrane, since ETR I was greatly suppressed (Fig. 3). The *PGR5* KD plants showed a stepwise increase in the redox state of the plastoquinone pool (high 1-qL) at every transition to the low-light phase. This increase indicates that during every high-light phase, the electron transport system of the *PGR5* KD plants accumulates excess reducing power, which the system is not capable of dissipating as heat. The cumulative strong reduction of the entire electron transport system under fluctuating light would cause a strong reducing burst at the acceptor side of PSI, as indicated by Y(NA) (Fig. 3), eventually leading to the photoinhibition of PSI (Figs 5 and 7). As a result, the *PGR5* KD plants decreased their CO_2_ assimilation rate via reductions in ETR I and ETR II (Fig. 3), and their growth was subsequently retarded under the fluctuating light conditions (Fig. 6). The similarity of these observations in rice *PGR5* KD to those in Arabidopsis *pgr5* mutants shows that plants with defects in PGR5 suffered from fluctuating light, with PSI as the primary target of photodamage and a stunted phenotype^8^,^9^. Therefore, the physiological function of the PGR5-dependent CEF pathway seems to be conserved among higher plants.

On the other hand, the NDH-defective mutant showed photosynthetic reactions almost identical to those in WT plants under constant high light (Fig. 4). However, the plastoquinone pool was reduced more (high 1-qL) at low light intensity than in the WT plants (Figs 2 and 4). The mutant showed a stepwise increase in the redox state of the plastoquinone pool at every transition to the low-light phase, as in the *PGR5 KD* plants. This increase indicates that during every high-light phase, the electron transport system of the NDH defective mutant accumulates excess reducing power (Fig. 4), which would cause photoinhibition of PSI (Figs 5 and 7), as in the *PGR5* KD plants (Figs 3,5 and 7). As a result, the NDH-defective mutant decreased its CO_2_ assimilation rate via reductions in ETR I and ETR II (Fig. 4), and thus its growth, under fluctuating light (Fig. 6). Previous studies showed that even the complete absence of the NDH complex in Arabidopsis did not suppress growth under fluctuating light^9^,^10^. Thus, there is an interspecific difference between Arabidopsis and rice in the physiological function of NDH-dependent CEF-PSI. Although substantial progress has been made in understanding the structure and enzyme activity of the chloroplast NDH complex, the physiological significance of the complex remains to be clarified. Disturbed electron transfer parameters and reduced plant growth and grain production of an NDH-defective mutant of rice highlight the physiological significance of the NDH-dependent CEF-PSI under non-optimal growth conditions, including low temperature^15^ and low light^25^. Our results highlight the importance of NDH-dependent CEF-PSI under fluctuating light in rice.

In both the *PGR5* KD plants and the NDH-defective mutant, the PSI reaction center remained fully reduced during the high-light phase, whereas in the WT plants, it became normally oxidized at transition to the high-light phase (Figs 3 and 4). This change implies that the photosynthetic machinery in the WT plants is capable of decreasing the electron flow to PSI during the high-light phase, which seems to be essential to preventing the over-reduction of PSI and to keeping the reduction level of the entire electron transport chain low enough during the subsequent low-light phase. In addition, when the low-light phase is followed by a high-light phase, rapid oxidation of the highly reduced electron transport chain (i.e., mainly the plastoquinone pool) in the two mutants exceeds the capacity of immediate PSI electron acceptors, as indicated by the PSI acceptor side limitation in these mutants (Figs 3 and 4). Such an excess of electrons could damage PSI under fluctuating light. Our results emphasize the importance of the PGR5-dependent and NDH-dependent CEF-PSI for sustaining photosynthesis and plant growth under fluctuating light in rice.

The physiological functions of state transition and PGR5-PGRL1-dependent CEF-PSI have been clarified in Arabidopsis under fluctuating light to represent natural conditions^8^,^9^,^10^. In particular, the PGR5-PGRL1-dependent CEF-PSI plays a central role in the regulation of linear electron transport via the downregulation of the Cyt *b*_6_*/f* complex^26^,^27^,^28^. It is still open for discussion how the PGR5-PGRL1 proteins regulate the rate of linear electron transport, since the exact molecular function of PGR5 and PGRL1 has been a topic of debate^29^. Here, we revealed similar phenotypes in the rice *crr6* mutant and *PGR5* KD rice under fluctuating light. This discrepancy with the phenotype in the Arabidopsis NDH-knockout mutants could be explained by the different physiological significance of NDH-dependent CEF-PSI in rice and Arabidopsis. Notably, a similar phenotype under fluctuating light was observed in two rice mutants defective in different CEF-PSI pathways. The most straightforward explanation for this observation is that both PGR5-PGRL1 and chloroplast NDH regulate the rate of linear electron transport by controlling the activity of the Cyt *b*_6_*/f* complex via CEF-PSI, as both pathways contribute to creating the proton motive force in the light^30^. It is also likely that both CEF-PSI pathways prevent the over-reduction of the stroma by balancing the ATP/NADPH production ratio in photosynthesis.

## Materials & Methods

### Plant materials and growth conditions

The rice *crr6* mutant (defective in the *OsCRR6* gene^15^) and its WT (*Oryza sativa* cv. Hitomebore), and the knockdown (KD) rice of the *OsPGR5* gene^11^ and its WT (*Oryza sativa* cv. Nipponbare) were grown in soil in an environmentally controlled growth chamber. Each seedling was planted in a 1.3-L plastic pot with 1.0 g of a slow-release fertilizer (Temairazu; Co-op Chemical Co., Ltd., Tokyo, Japan). The chamber was operated with a day/night temperature of 28/23 °C, a relative humidity of 65%, a PPFD of 500 μmol m^–2^ s^–1^, a 14-h photoperiod, and a CO_2_ concentration of 400 μmol mol^–1^.

For the analysis of growth under fluctuating light, plants grown as above for 30 days were transferred to either constant high light (800 μmol m^–2^ s^–1^) or fluctuating light (150 μmol m^–2^ s^–1^ for 10 min and 800 μmol m^–2^ s^–1^ for 10 min) for another 40 days.

### Analysis of gas exchange, chlorophyll fluorescence and P700 measurements

Gas exchange, chlorophyll fluorescence, and P700 redox state were measured simultaneously with a GFS-3000 and a Dual-PAM-100 measuring systems (Walz, Effeltrich, Germany) in the uppermost, fully expanded new leaves of 60- to 80-day-old plants as described^15^. After leaves were dark-adapted for 30 min, a saturating pulse was applied to obtain the maximum fluorescence and the maximum change in P700. Several photosynthetic parameters were measured every 20 s at a CO_2_ concentration of 400 μmol mol^–1^ under either constant high light or fluctuating light. The quantum yield of PSI (Y(I)) was calculated as Y(I) = 1 – Y(ND) – Y(NA), where Y(ND) corresponds to the fraction of P700 that is already oxidized by actinic light and Y(NA) corresponds to the fraction of P700 that are closed owing to acceptor side limitation. Since it has been reported that the signal of P700 is slightly affected by plastocyanin-dependent signal^31^, the parameters of P700 may be slightly affected although it should not be so significant. The quantum yield of photosystem II [Y(II) = (*F*_m_′–*F*′)/*F*_m_′], photochemical quenching [qP = (*F*_m_′–*F*′)/(*F*_m_′–*F*_o_′)], non-photochemical quenching [NPQ = (*F*_m_–*F*_m_′)/*F*_m_′], and the fraction of PSII centers in the open state (with plastoquinone oxidized) [qL = qP × (*F*_o_′/*F*′)] were calculated. The electron transport rate (ETR) was calculated as ETR I (or ETR II) = 0.5 × abs I × Y(I) (or Y(II)), where 0.5 is the fraction of absorbed light reaching PSI or PSII, and abs I is absorbed irradiance taken as 0.84 of incident irradiance.

### Analysis of photoinhibition

The leaves were placed in a temperature-controlled chamber at a CO_2_ concentration of 400 μmol mol^–1^ and a relative humidity of 65% in the Dual-PAM-100 and GFS-3000 measuring systems. Photoinhibition was analyzed immediately after the measurements of photosynthesis under constant high light or fluctuating light (Figs 3 and 4). The leaves were exposed to constant high light (1500 μmol photons m^–2^ s^–1^) for 5 h or fluctuating light (high light at 1500 μmol m^–2^ s^–1^ for 10 min and low light at 200 μmol m^–2^ s^–1^ for 10 min) for 5 h. The maximum level of the P700 signal (full oxidation of P700) in the dark and the maximum quantum yield of PSII (*F*_v_/*F*_m_) after dark incubation for 30 min were measured before and after the 5 h light treatment.

### Quantifications of photosynthetic components and Immunoblot analysis

Immediately after the measurements of gas exchange, leaf samples were taken, immersed in liquid nitrogen, and stored at –80 °C. The frozen leaf samples were ground in liquid nitrogen and homogenized in an extraction buffer 12. Contents of leaf nitrogen, chlorophyll, and rubisco were quantified^12^,^25^.

## Additional Information

**How to cite this article**: Yamori, W. *et al*. A physiological role of cyclic electron transport around photosystem I in sustaining photosynthesis under fluctuating light in rice. *Sci. Rep.* **6**, 20147; doi: 10.1038/srep20147 (2016).

## Supplementary Material

Supplementary Information

## Figures and Tables

**Table 1:** Physiological components of photosynthesis.

**Parameter**	**Total N**	**Rubisco**	**Chl**	**Chl** ***a/b***
	**(mmol m**^**−2**^)	**(μmol m**^**−2**^)	**(mmol m**^**−2**^)	
WT (Nipponbare)	105 ± 4	3.46 ± 0.18	0.60 ± 0.07	3.82 ± 0.06
*PGR5* KD	100 ± 5	3.32 ± 0.24	0.58 ± 0.09	3.75 ± 0.09
WT (Hitomebore)	109 ± 6	3.62 ± 0.14	0.63 ± 0.06	3.92 ± 0.08
*crr6*	106 ± 5	3.69 ± 0.18	0.61 ± 0.02	3.85 ± 0.09

Contents of total nitrogen (Total N), Rubisco, and chlorophyll (Chl) were quantified. Data represent means ± SE, *n* = 5 or 6. Tukey–Kramer multiple comparison test showed no significant differences among samples (*P* < 0.05).
