# Supplementary material for: A physiological role of cyclic electron transport around photosystem I in sustaining photosynthesis under fluctuating light in rice
Source: Sci Rep. 2016 Feb 2;6:20147. doi: 10.1038/srep20147 (PMC4735858; doi:10.1038/srep20147)
Supplement: Supplementary Information [file srep20147-s1.pdf]

Title

A physiological role of cyclic electron transport around photosystem I in sustaining photosynthesis under fluctuating light in rice

Wataru Yamori[1,4], Amane Makino[2, 5] and Toshiharu Shikanai[3,5]

[1]Center for Environment, Health and Field Sciences, Chiba University, 6-2-1 Kashiwa-no-ha, Kashiwa, Chiba 277-0882, Japan

[2]Department of Applied Plant Science, Graduate School of Agricultural Science, Tohoku University, 1-1 Tsutsumidori-Amamiyamachi, Aoba-ku, Sendai 981-8555, Japan

[3]Department of Botany, Graduate School of Science, Kyoto University, Sakyo-ku, Kyoto 606-8502, Japan

[4]PRESTO, Japan Science and Technology Agency (JST), 4-1-8 Honcho, Kawaguchi, Saitama 332-0012, Japan

[5]CREST, JST, 4-1-8 Honcho, Kawaguchi, Saitama 332-0012, Japan

Supplemental Figure 1

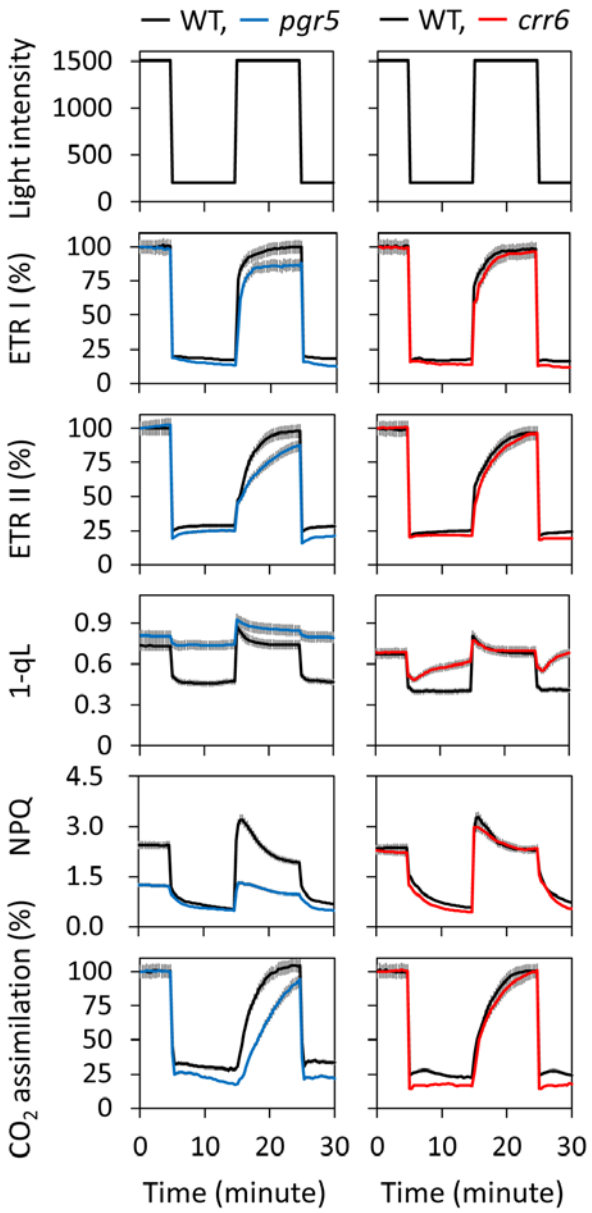

Time course of photosynthetic responses after changes in light intensity in *PGR5*-knockdown plants, *crr6* mutant plants, and WT plants. The same fluctuating-light regime as used for the data in Figures 2 was used. Also, abbreviations are the same as those in Figure 2. ETR I, ETR II and CO_2 assimilation rate were shown as relative values to the rate of each photosynthetic parameter at the steady-state photosynthesis. The absolute values for each photosynthetic parameter can be observed in Figures 3 & 4. The graphs compare *PGR5* KD plants with their WT (*Oryza sativa* cv. Nipponbare), and *crr6* mutant plants with their WT (*Oryza sativa* cv. Hitomebore). Data represent means ±SE, n = 5~6.

Supplemental Figure 2

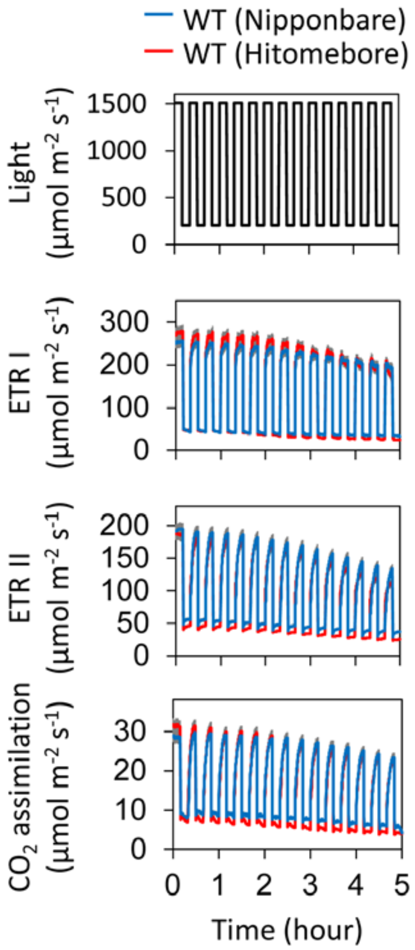

Responses of photosynthetic parameters to fluctuating light in WT (‘Nipponbare’) plants and WT (‘Hitomebore’) plants. Photosynthetic parameters were monitored at a CO_2 concentration of 400 µmol mol^{-1} under fluctuating light (200 µmol m^{-2} s^{-1} for 10 min and 1500 µmol m^{-2} s^{-1} for 10 min) for 5 h. Abbreviations are the same as in Figure 2. Values are means ± SE, *n* = 5 or 6.

Supplemental Figure 3

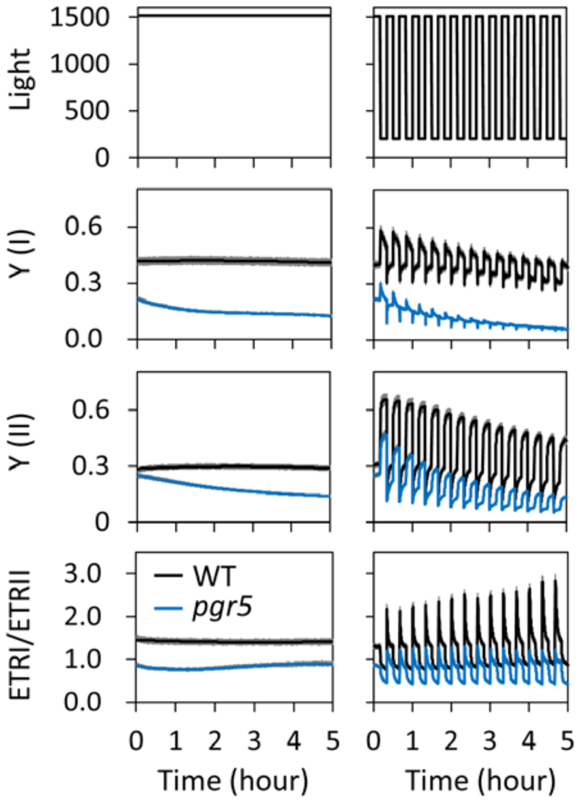

Responses of photosynthetic parameters to either constant high-intensity or fluctuating light in *PGR5*-knockdown and WT ('Nipponbare') plants. The quantum yield of PSI (Y(I)) and PSII (Y(II)), and also the ratio of ETRI/ETRII were monitored at a CO_2 concentration of 400 µmol mol^{-1} under either constant high-intensity light (1500 µmol photons m^{-2} s^{-1}) or fluctuating light (200 µmol m^{-2} s^{-1} for 10 min and 1500 µmol m^{-2} s^{-1} for 10 min) for 5 h were presented. The electron transport rate (ETR) was calculated as ETR I (or ETR II) = 0.5 × abs I × Y(I) (or Y(II)), where 0.5 is the fraction of absorbed light reaching PSI or PSII, and abs I is absorbed irradiance taken as 0.84 of incident irradiance (see, Materials & Methods). Values are means ± SE, n = 5 or 6.

Supplemental Figure 4

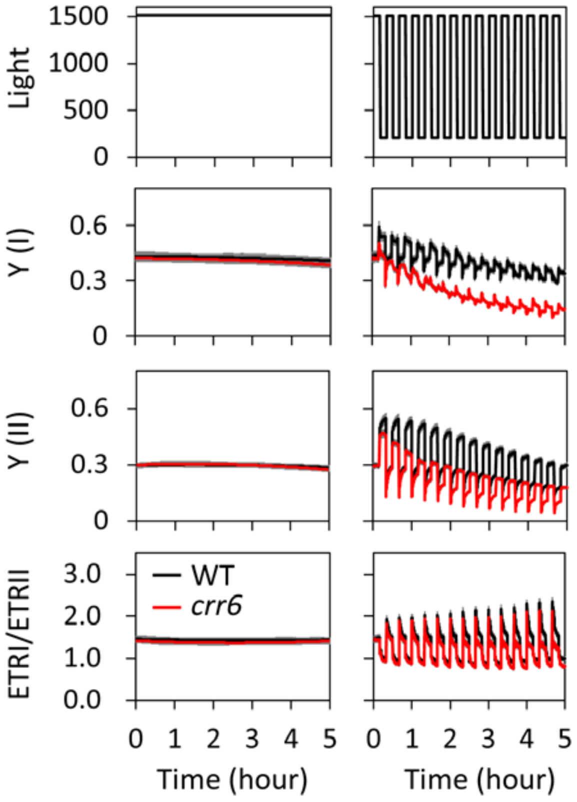

Responses of photosynthetic parameters to either constant high-intensity or fluctuating light in *crr6* mutant and WT ('Hitomebore') plants. The quantum yield of PSI (Y(I)) and PSII (Y(II)), and also the ratio of ETRI/ETRII were monitored at a CO_2 concentration of 400 μmol mol^{-1} under either constant high-intensity light (1500 μmol photons m^{-2} s^{-1}) or fluctuating light (200 μmol m^{-2} s^{-1} for 10 min and 1500 μmol m^{-2} s^{-1} for 10 min) for 5 h were presented. The electron transport rate (ETR) was calculated as ETR I (or ETR II) = 0.5 × abs I × Y(I) (or Y(II)), where 0.5 is the fraction of absorbed light reaching PSI or PSII, and abs I is absorbed irradiance taken as 0.84 of incident irradiance (see, Materials & Methods). Values are means ± SE, n = 5 or 6.

Supplemental Figure 5

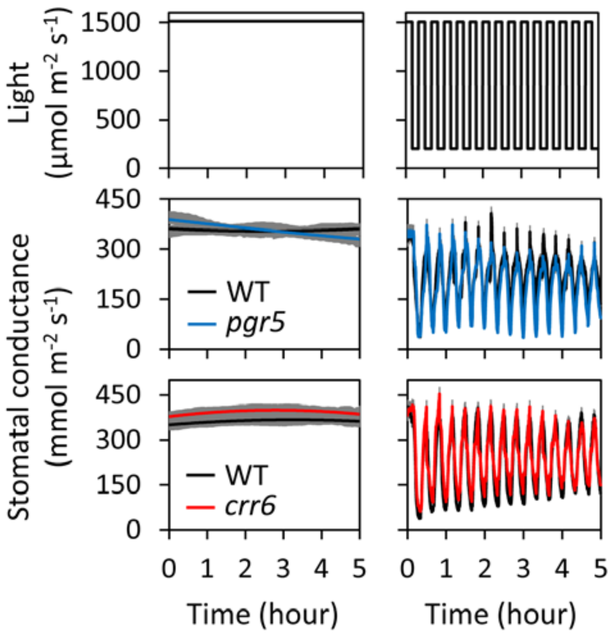

Response of the stomatal conductance at CO_2 concentration of 400 µmol mol^{-1} either to the constant high light or fluctuating light in *PGR5* KD plants, *crr6* mutant and the WT plants. The same constant high-light or fluctuating-light regime as used for the data in Figures 3 and 4 was used. Either constant high-light (1500 µmol photons m^{-2} s^{-1}) or fluctuating-light conditions fluctuating light (200 µmol m^{-2} s^{-1} for 10 min and 1500 µmol m^{-2} s^{-1} for 10 min) was applied to the leaf. The graphs compare *PGR5* KD plants with their WT (*Oryza sativa* cv. Nipponbare), and crr6 mutant plants with their WT (*Oryza sativa* cv. Hitomebore). Data represent means ±SE, n = 5~6.
